# Role of pentoxifylline in neonatal hypoxic ischaemic encephalopathy: a systematic review of animal studies

**DOI:** 10.1186/s42826-024-00228-0

**Published:** 2024-11-28

**Authors:** Florence Wong, Chandra Rath, Bhanu B. Gowda, Sanjay Patole

**Affiliations:** 1 Division of General Paediatrics, Armadale Kelmscott Memorial Hospital, Mount Nasura, WA 6112 Australia; 2 Perth Children’s Hospital, Nedlands, WA 6009 Australia; 3 King Edward Memorial Hospital, Subiaco, WA 6008 Australia; 4 School of Medicine, University of Western Australia, Nedlands, WA 6009 Australia

**Keywords:** Pentoxifylline, Hypoxic ischemic encephalopathy, Animal

## Abstract

We systematically reviewed the evidence from animal studies assessing the effects of pentoxifylline on neonatal hypoxic-ischemic encephalopathy (HIE). The PubMed, EMBASE, EMCARE, MEDLINE, Cochrane Library, and Google Scholar databases were searched for randomized and quasi randomized controlled trials (RCTs) in December 2023 to determine the effects of pentoxifylline in animal models of HIE. The quality of the included studies was assessed via the SYRCLE risk of bias (ROB) tool. The certainty of evidence was assessed via the GRADE methodology. All seven included studies (n = 248) involved a rat HIE model in which pentoxifylline (25–150 mg/kg) was administered intraperitoneally. The majority had unclear ROB. All the studies reported a protective effect of pentoxifylline on HIE-induced organ injury. Mortality was comparable at pentoxifylline doses between 25 and 75 mg/kg but higher at 150 mg/kg than in the control group. Three studies reported macroscopic changes in HIE-affected organs. There was a significant reduction in cerebral infarction (40 and 75 mg/kg), hippocampal atrophy, and visible gut injury (60 mg/kg). A significantly lower number of Caspase 3 immunoreactive cells and necrotic cells were observed at the 60 mg/kg dose, whereas the 100 mg/kg dose had a deleterious effect. Three other studies reported significantly reduced levels of proinflammatory markers including IL-6 and TNF-alpha. Current evidence (with low uncertainty) from a rat model suggests that pentoxifylline has the potential to improve mortality and attenuate organ injury following HIE. Adequately powered, well-designed human RCTs are needed to confirm our findings.

## Background

Hypoxic ischemic encephalopathy (HIE) is a potentially serious birth complication affecting newborn infants [1]. The pathogenesis of brain injury in HIE involves inadequate cerebral blood flow due to a hypoxic-ischemic event in the prenatal, intrapartum, or postnatal period. The clinical manifestations of HIE indicate brain dysfunction. The prevalence of HIE is 0.5 to 3/1000 live births in high-income countries (HICs) [2, 3] and 10 to 40/1000 live births in lower- and middle-income countries (LMICs) [4–6]. HIE is associated with significant mortality (25%) and morbidity (20%) [7, 8] with over 90% of HIE-related deaths and impairments occurring in low-resource settings [4, 9].

Importantly, despite therapeutic hypothermia (TH), the only established treatment for this condition [10, 11], 30–50% of infants with moderate-to-severe HIE are at risk of death or significant disability [12]. TH is an accepted treatment for HIE in the HIC setting; however, the results of a large randomized controlled trial (RCT) have questioned its safety in LMICs [13]. Mortality was significantly greater in the TH group in this trial. The authors recommended that TH should not be offered as a treatment for HIE in LMICs, even if tertiary neonatal intensive care facilities are available [13].

Given the global burden of HIE, the limited therapeutic options, and the increased risk of mortality associated with TH in LMICs, there is an urgent need for new strategies (standalone or adjuvant to TH) to improve the outcomes of HIE. Pentoxifylline is an immunomodulator with anti-inflammatory properties and is a nonselective phosphodiesterase inhibitor. It reduces the synthesis of tumour necrosis factor alpha, interleukin-1, interleukin-6, and interferon gamma through adenosine A2A receptor-mediated pathways and may reduce tissue damage during the cytokine storm [14]. Pentoxifylline lowers blood viscosity and improves microcirculation and tissue perfusion through its antiaggregatory and vasodilatory actions. It also inhibits white cell activation and oxidative injury [15]. Considering the role of hypoxia–ischemia-reperfusion and oxidative injury in this condition, pentoxifylline has the potential to be an effective early intervention in neonates with HIE [16, 17].

To our knowledge, only one small quasi-RCT has assessed the effects of pentoxifylline in neonatal HIE [18]. This trial reported a reduction in mortality without adverse effects in the intervention arm [18]. Although encouraging, these results are inadequate for proceeding with large well-designed RCTs to assess the effects of pentoxifylline in neonatal HIE. Critical assessment of the evidence from animal studies (the gold standard for guiding clinical research) is equally or perhaps more important in this context [19]. Hence, we aimed to review the evidence from animal studies assessing the effects of pentoxifylline in HIE.

## Main text

The systematic review centre for laboratory animal experimentation (SYRCLE) protocol [20] and Cochrane guidelines were used to conduct this systematic review which was reported according to the Preferred Reporting Items for Systematic Reviews and Meta-analysis (PRISMA) statement [21]. It was registered on the Open Science forum (https://doi.org/10.17605/OSF.IO/27GE4).

The PubMed, EMBASE (through OVID), EMCARE (through OVID), MEDLINE (through OVID), Cochrane Library and Google Scholar databases were searched (from inception until December 2023) independently by two reviewers. The strategy of having two experienced reviewers conduct an independent literature search was used to enhance the precision of study selection [22].

The ClinicalTrials.gov website was searched to identify ongoing studies. The Gray literature was searched in the Mednar (http://mednar.com/mednar/desktop/en/search.html) database*.* Abstracts from the meetings of the Paediatric Academic Society (PAS), and conference proceedings including the Perinatal Society of Australia and New Zealand (PSANZ) and the European Academy of Paediatric Societies were searched and reviewed beginning in 2017. PubMed was searched via the following keywords: ("pentoxifylline"[MeSH Terms] OR "pentoxifylline"[All Fields] OR "pentoxyfylline"[All Fields]) AND ("hypoxic ischaemic encephalopathy"[All Fields] OR "hypoxia ischemia, brain"[MeSH Terms] OR ("hypoxia ischemia"[All Fields] AND "brain"[All Fields]) OR "brain hypoxia–ischemia"[All Fields] OR ("hypoxic"[All Fields] AND "ischemic"[All Fields] AND "encephalopathy"[All Fields]) OR "hypoxic ischemic encephalopathy"[All Fields]). Similar terms were used to search other databases. We manually searched the reference lists of relevant articles without applying time or language restrictions. The references identified from the database search were exported to EndNote software. Duplicate articles were removed, and the full texts of eligible studies were obtained after the abstracts were read. The full text articles were read independently by two reviewers to assess their suitability for inclusion. Discrepancies in study selection were resolved by discussion with a third reviewer.

RCTs and quasi-RCTs in animal models meeting the following criteria were eligible for inclusion: (1) use of a validated animal model of neonatal HIE (2); evaluation of pentoxifylline as an experimental intervention for HIE (3); and comparison of pre stated clinical and laboratory outcomes between the experimental and control groups.

Observational and in vitro studies, studies that did not have controls and studies that used unvalidated models were excluded.

The outcomes included (1) mortality from HIE; (2) macroscopic, microscopic, and immune-histological findings in any organ; (3) laboratory markers of inflammation and oxidative injury; (4) short and long-term outcomes; and (5) other outcomes reported by the study authors.

A pre prepared standardized form was used to extract data. Two reviewers independently completed the data collection forms. The data on primary and secondary outcomes were abstracted from the included studies. We recorded the odds ratios (ORs) or risk ratios (RRs) for the dichotomous outcomes if available in the included studies. The quality of the included animal RCTs was assessed using the SYRCLE risk of bias (ROB) tool [20]. The certainty of evidence was assessed using the Grading of Recommendations, Assessment, Development, and Evaluations (GRADE) methodology and classified into one of the four categories: high, moderate, low and very low [23]. In case of discrepancies, group discussions were held involving all authors for reaching consensus.

We planned to conduct a meta-analysis via Review Manager v5.4 (Cochrane Collaboration, Nordic Cochrane Centre, Copenhagen, Denmark) via a random effects model (DerSimonian and Laird) to anticipate heterogeneity. The effect size was expressed as the RR and 95% confidence interval (CI) for dichotomous outcomes and the mean difference and 95% CI for continuous outcomes. We used raw numbers to calculate the RRs for pooling if the included studies did not provide this information. Qualitative synthesis was planned if meta-analysis was not possible. We planned to assess heterogeneity via the I^2^ statistic. The I^2^ results were interpreted as follows: 0–40%: might not be important; 30–60%: may represent moderate heterogeneity; 50–90%: may represent substantial heterogeneity; and 75–100%: may represent considerable heterogeneity (Cochrane Handbook) [24]. We planned to explore heterogeneity by considering differences in variables such as HIE model features, sample size and power estimation, protocol for pentoxifylline (dose, route, duration, prophylaxis vs treatment), comparator (no pentoxifylline or placebo), standardization of histopathological findings (e.g., validated scoring system), and pre stated vs post-hoc outcomes. We planned a sensitivity analysis after excluding studies with high ROB.

**Fig. 1:**
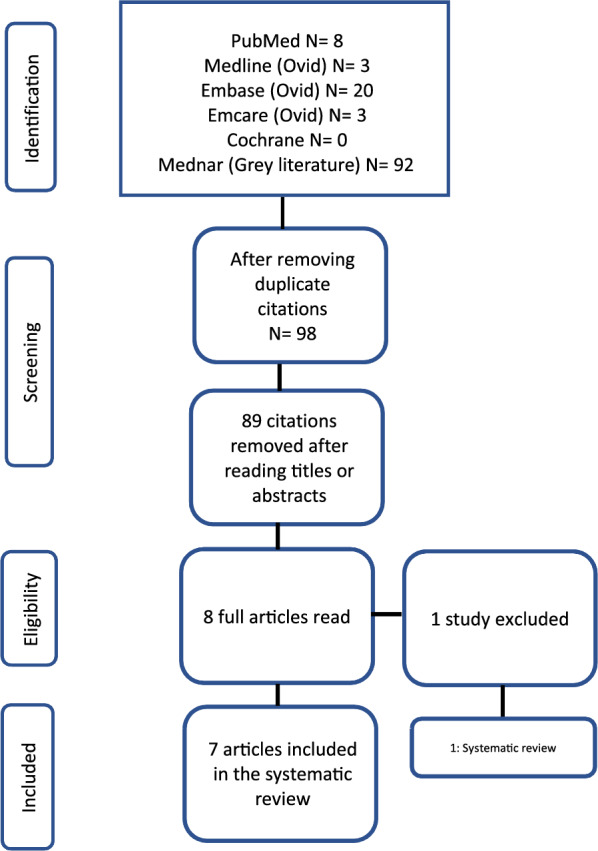
PRISMA flow chart for study selection

A PRISMA flow chart of the screening and selection of studies is shown in Fig. 1. The initial search identified 98 articles, 7 of which [25–31] (n = 248) were included after applying the selection criteria. One systematic review was excluded [32]. All included studies were RCTs or quasi-RCTs that evaluated pentoxifylline in an animal model of HIE (Table 1). The ROB was generally unclear for the majority of the included studies. The level of evidence was deemed low, mainly because of the uncertain ROB of the included studies.

**Table 1:** Characteristics of the studies included in the systematic review

Study id	Methods	Outcomes					Author’s conclusions
		mortality	Macroscopic finding	Microscopic finding/immunohistochemistry	Inflammatory and oxidative stress	Other	
(1) Kim [25] Korea	Animals: D7 rats Study design: Quasi RCT Model: Right carotid ligation followed by 3 h of exposure to 8% O2 Route of administration of Pentoxifylline (P): Intraperitoneal Sample and tests: Group 1 (n = 4): Neck incision and I and H (control) Group 2 (n = 3): Ischemia Hypoxia + P group: 40 mg/kg/dose of P immediately before and after hypoxia Rats euthanised: 4 h post hypoxia	NA	NA	NA	In group 2 IL-1beta and TNF alpha mRNA expression were attenuated at 4 h post hypoxia- ischemia	NA	Pretreatment with P reduces the incidence and severity of hypoxic-ischemic injury. P attenuated the expression of IL-1beta and TNF-alpha gene on hypoxic-ischemic injury in immature rat brain
(2) Eun [26] Korea	Animals: D7 Sprague–Dawley rats Study design: RCT Model: Right carotid ligation followed by 2.75–3.25 h of exposure to 8% O2 Route of administration of P: Intraperitoneal Sample and tests: Group 1: P 25 mg/kg/dose immediately before and after hypoxia (n = 5) Group 2: P 75 mg/kg/dose immediately before and after hypoxia (n = 11) Group 3: P 150 mg/kg/dose immediately before and after hypoxia (n = 5) Group 4: P 40 mg/kg/dose immediately before and after hypoxia (n = 13) Group 5 (Rescue): P 40 mg/kg/dose immediately and 3 h after hypoxia (n = 8) Rats euthanised: Day 12	Group 1: 1/5 (20%) Group 2: 2/11(18%) Group 3: 5/5 (100%) Group 4: 3/13 (23%) Group 5: 0/8 (0%) Control (before and after group): 3/27 (11%) Rescue control: 0/10 (0%)	Incidence of no cerebral infarction: Group 1: 1/5 Group 2: 8/11 Group 3: NA Group 4: 9/13 Group 5 (Rescue): 5/8 Control (Rescue): 3/10 Mean percent reduction in right cerebral hemisphere areas, in comparison to intact left-sided regions [100X(L-R)/L]: % reduction expressed in mean ± SD Group 1: Anterior 12 ± 7.5, Posterior 16.7 ± 10.8 Group 2*: Anterior 3.2 ± 4.8, posterior 3.1 ± 5.8, Group 3: NA, Group 4*: Anterior 4.5 ± 5.8, posterior 4.5 ± 4.7 Control (Before and after): Anterior 14.3 ± 10.4, posterior 16.8 ± 12.1 Group 5 (Rescue): Anterior 9.8 ± 11.9, posterior 12.4 ± 11.5 Control (Rescue): Anterior 13.4 ± 11.7, posterior 20.5 ± 13.3 Special regional area volume estimation only in Group 5 (Rescue): Bilateral cortical, striatal, and hippocampal volumes damage was significantly less severe compared to controls	Only Group 5 (Rescue) information available: Cortical infarctions were less extensive compared to controls	NA	NA	Pretreatment with P could be clinically relevant in settings in which an increased risk of cerebral ischemia can be anticipated, such as in infants undergoing surgery to correct CHD. post hypoxic-ischemic treatment with P resulted in only a modest reduction in cortical damage, without an overall reduction in incidence of infarction
(3) Kalay [27] Turkey	Animals: D7 Wistar rats Study design: RCT Model: Left carotid ligation followed by 2 h of exposure to 8% O2 Route of administration of P: Intraperitoneal Sample and tests: Group 1 (n = 16): I and H and saline control group Group 2 (n = 16): I and H + P group: 60 mg/kg/dose of P immediately after hypoxia Group 3 (n = 8): Only neck incision but no I and H control group. No intervention Rats euthanised: 4 to 24 h after intervention	NA	NA	NA	TNF Alpha m- RNA, IL-1b mRNA, Caspase 3 (Left cerebral tissue): Significantly reduced in Group 2 compared group 1 (*P* < 0.01 for 4 and 24 h)	NA	P may reduce brain damage due to I and H injury
(4) Kalay [28] Turkey	Animals: D7 Wistar rats Study design: RCT Model: Left carotid ligation followed by 2 h of exposure to 8% O2 Route of administration of P: Intraperitoneal Sample and tests: Group 1 (n = 8): Only neck incision but no I and H(control) Group 2(n = 8): I and H and saline control group Group 3 (n = 8): IH + P group: 60 mg/kg/dose of P immediately after hypoxia Rats euthanised: 24 h after drug administration	Group 1: 0/8, group 2: 0/8, group 3: 0/8	Intestinal injury in IH model: Dark pink/purple discolouration of gut: Group 1: 0/8, group 2: 4/8, group 3: 2/8	Group 1: 0/8 Group 2: 8/8 (4 had 4th level damage, 3 had 3rd level damage and 1 had 1st level damage) Group 3: 6/8 (1 had 3rd level damage, 2 had 2nd level damage and 3 hade level 1 damage) Mean Chiu/Park histopathological scoring levels reduced significantly at 24th hour in the group 3 compared to group 2 (*p* < 0.05 for 24 h)	NA	NA	P significantly attenuated hypoxia ischemia induced intestinal injury in neonatal rat model of IH encephalopathy

(5) Park [29] Korea	Animals: D7 Sprague–Dawley rats Study design: RCT Model: Right carotid ligation followed by 90 min of exposure to 8% O2 Route of administration of P: Intraperitoneal Sample and tests: Group 1 (n = 10): Sham operation only Group 2 (n = 10): I and H control Group 3 (n = 10): Induced IH and 50-mg/kg P-treated (Once a day for 7 days) Group 4 (n = 10): IH induced and 100-mg/kg P-treated (Once a day for 7 days) Rats euthanised: Immediately after determining the latency in the step-down avoidance task	NA	NA	NA	Caspase-3 expression in the hippocampal CA1 region was enhanced by perinatal hypoxic-ischemic injury (*P* < 0.05), and P suppressed this increase (*P* < 0.05)	Short term memory disrupted by hypoxia ischemia was significantly alleviated by P (*p* < 0.05). Hippocampal cAMP level, DNA fragmentation was reduced by P (*p* = < 0.05). P suppressed Bax expression (*P* < 0.05) and enhanced Bcl-2 expression (*P* < 0.05)	P ameliorated perinatal IH in rat pups. This alleviating effect could be ascribed to the inhibition apoptosis due to increased cAMP production
(6) Halis [30] Turkey	Animals: D7 Wistar male rats Study design: RCT Model: Right carotid ligation followed by 2 h of exposure to 8% O2 Route of administration of P: Intraperitoneal Sample and tests: Group 1 (n = 5): Sham operation only Group 2(n = 6): I and H control Group 3 (n = 6): Induced IH and 60-mg/kg P-treated (immediately after hypoxia and then after 2 h) Group 4 (n = 6): IH induced and 100-mg/kg P-treated (immediately after hypoxia and then after 2 h) Rats euthanised: 24 h after hypoxia–ischemia	Group 1: 1/5 (20%) Group 2: 2/6 (33%) Group 3: 2/6 (33%)	NA	Healthy neurons, caspase 3 positive and necrotic cells: The number of healthy normal neuron in the left in group 4 were significantly lower than group 1, 2 and 3 (P < 0.05). In terms of Caspase-3 immunoreactive cells, no difference was found between group 2, 3 and 4. However, in the CA region of the hippocampus the elevation caspase-3 immunoreactive cells in group 3 was significantly lower that group 2. The numbers of necrotic cells in the parietal cortex and dentate gyrus were significantly higher in the group 2 and 4 than in group 3 (P < 0.05), and their numbers in the CA regions of the hippocampus was significantly lower in the group 3 group than in group 2 (*P* < 0.05)	NA	NA	P administration after IH reduces neuronal apoptosis and necrosis. Therefore, P may be an effective drug to use to decrease the severity of neonatal IH brain injury
(7) Halis [31] Turkey	Animals: D7 Wistar male rats Study design: RCT Model: Right carotid ligation followed by 2 h of exposure to 8% O2 Route of administration of P: Intraperitoneal Sample and tests: Group 1 (n = 17): Naïve rats with no intervention Group 2 (n = 13): Sham operation only Group 3 (n = 16): I and H control Group 4 (n = 20): Induced IH and 60-mg/kg P-treated (immediately after hypoxia and then after 2 h) Group 5 (n = 11): IH induced and 100-mg/kg P-treated (immediately after hypoxia and then after 2 h) Rats euthanised: One day after cognitive assessment (Usually, d77 to 81)		Hippocampal atrophy following unilateral carotid artery ligation could be partly conserved by P at dose of 60 mg, but not by dose of 100 mg	NA	NA	Carotid artery ligation resulted in a prolongation of path length to reach the hidden platform and that P at a dose of 60 mg, but not at 100 mg, prevented this impairment. Carotid artery ligation results in impairment of memory retrieval and that low dose of P (60 mg) is more effective than high dose (100 mg) to prevent this impairment	Unilateral IH brain injury in a neonatal rodent model is associated with cognitive deficits, and that low dose P treatment is protective against spatial memory impairment

P, Pentoxifylline; NA, Not available; IL, Interleukin; TNF, Tissue necrosis factor; I and H, Ischemia and hypoxia; CHD, Congenital heart disease; cAMP, Cyclic adenosine monophosphate; RCT, Randomized controlled trial; HIE, Hypoxic ischemic encephalopathy; CA, Cornu Ammonis

^*^Significantly better than the control

All the included studies used a rat HIE model. Hypoxia was induced by ligating the carotid artery and exposing the animals to 8% oxygen for 2 to 3 h. The dose of pentoxifylline used in the included studies ranged from 25 to 150 mg/kg. The route of administration was intraperitoneal in all included studies. All included studies reported the protective effect of pentoxifylline on HIE induced organ injury.

Two of the included studies used pentoxifylline for prophylaxis as well as treatment, whereas the other five used pentoxifylline as a treatment after inducing hypoxia [25, 26]. Three of the included studies reported mortality rates ranging from 0 to 100% [26, 28, 30]. One of the included studies reported 100% mortality at a dose of 150 mg/kg pentoxifylline [26]. Mortality rates were comparable at doses between 25 and 75 mg/kg. Another study used a dose of up to 100 mg/kg and reported comparable mortality between the treatment and control groups [30].

A dose of 60 mg/kg was associated with no significant difference (0% vs. 0%) in mortality between the intervention and control groups in one of the included studies [28].

Macroscopic findings of HIE-induced organ injury were reported in three of the included studies [26, 28, 31]. A significant reduction in cerebral infarction was reported at doses of 40 and 75 mg/kg but not at 25 mg/kg [26]. In another study, a dose of 60 mg/kg pentoxifylline, but not 100 mg/kg pentoxifylline, resulted in a partial reduction in hippocampal atrophy [31]. Similarly, a dose of 60 mg/kg resulted in a significant reduction in HIE-related visible gut injury compared with that of the control [28]. Compared with control treatment, treatment with 60 mg/kg but not 100 mg/kg pentoxifylline resulted in significantly fewer caspase 3 immunoreactive cells and necrotic cells [30]. The number of healthy neurons was significantly lower in the 100 mg/kg group than in the control and 60 mg/kg groups [30]. Similarly, in another study, Compared with control treatment, pentoxifylline at a dose of 60 mg/kg resulted in a significant reduction in brain damage, as assessed by histopathological grading(28). Three studies reported a significant reduction in proinflammatory markers such as interleukins, tumour necrosis factor alpha and Caspase 3 in pentoxifylline-treated animals compared with control animals [25, 27, 29]. Two studies described the attenuating effects of pentoxifylline on impaired short-term memory and cognitive deficits induced by HIE [29, 31].

Our systematic review provides preliminary evidence supporting further studies of pentoxifylline as a potential treatment for neonatal HIE. The limitations of our findings include the small number of studies and cumulative sample size (7 studies, 248 animals), the unclear ROB in the majority of the included studies, heterogeneity in their approach (prophylaxis and/or treatment) and doses (25–150 mg/kg) of pentoxifylline. However, the fact that all included studies were RCTs or quasi-RCTs and utilized the widely accepted rat model of HIE is assuring [33]. The methodologies used in all included studies were comparable, involving the induction of hypoxia by ligating the carotid artery and exposing the animals to 8% oxygen for 2 to 3 h and the intraperitoneal administration of pentoxifylline. Importantly, all the included studies reported the protective effect of pentoxifylline on HIE-induced organ injury.

The limitations of the rat model of HIE need to be discussed. HIE results from severe systemic oxygen deprivation and compromised cerebral blood flow during the prenatal or perinatal period. The animal model used in the included studies was the Levine-Rice rat model to replicate neonatal HIE, which involves the induction of ischemia by carotid artery ligation and hypoxia by exposure to 8% oxygen [34]. The hypoxic-ischemic brain injury induced by this method predominantly affects the middle cerebral artery territory. Importantly, while hypoxemia is global in this model, ischemic injury can be localized with a variable degree of infarction. Consequently, varying degrees of infarction hinder the ability to study selective neuronal cell death, resulting in a lack of consistent and reproducible alignment between the physiological insult and manifestation of brain injury. Additionally, the cerebral cortex of rodents is lissencephalic and lacks the distinct regional divisions found in the human brain, limiting the ability to study specific anatomical areas and conduct separate biochemical analyses of white and grey matter. All the included studies used 7-day-old rats, whose brain development may correspond to a slightly premature neonate [33]. Ten-day-old rats may better mimic the neurobehavioral characteristics and pathological changes observed in full-term human neonates with HIE [35]. Therefore, future studies should consider the use of 10-day-old rats to more accurately model human HIE. Importantly, rat models have other inherent limitations, including difficulties in monitoring multiple organ dysfunction, as well as variations in gender-specific brain histology, behavioural outcomes, and responses to treatment [36]. Although rodent models of neonatal HIE have enhanced our understanding of the cellular mechanisms involved in neural injury in the developing brain, these models fall short in accurately simulating perinatal human brain injury, which is complex and influenced by various factors, including the timing and duration of the insult. Studies involving larger animals (e.g., piglets and primates) may help overcome some of the limitations of rodent models [37]. Acknowledging the limitations of animal models which are often viewed as the gold standard for informing clinical research, is important in this context [38–40].

The need for robust preclinical studies evaluating pentoxifylline as a treatment for HIE is further justified by the unique properties of the drug in relation to the pathophysiology of this condition and the evidence regarding its use in preterm infants [41, 42].

Given our findings, the importance of dose–response studies of pentoxifylline in the context of critical outcomes such as mortality and brain injury in HIE cannot be overemphasised. As an outcome, mortality competes with long-term survival without brain injury. Considering the difficulties in assessing long-term neurodevelopmental outcomes in animal models of HIE, the differences in histopathological findings suggestive of brain injury at different doses of pentoxifylline are important to note when designing future studies [43]. The evidence from the studies included in our systematic review and those in preterm infants with late onset sepsis (LOS) or necrotizing enterocolitis (NEC ≥ Stage II) will be helpful in this context.

Pammi et al. reported a systematic review to assess the effectiveness and safety of intravenous pentoxifylline as an adjunct to antibiotic therapy in reducing mortality and morbidity in neonates with suspected or confirmed sepsis or NEC [41]. All six RCTs (n = 416) included in their review assessed the effects of pentoxifylline in neonatal sepsis patients. The results (low-certainty evidence) suggested that the use of pentoxifylline as an adjuvant treatment may reduce mortality and shorten the hospital stay without adverse effects. Notably none of the included RCTs reported any adverse effects associated with pentoxifylline in any of the comparisons. The authors emphasised the need for further research highlighting the safety of intravenous pentoxifylline in critically ill preterm infants with sepsis or NEC [44]. The only clinical trial reported in the literature included 20 term neonates with moderate to severe HIE. The participants were subjected to modest hypothermia and randomly assigned to receive either pentoxifylline or an equivalent dose of placebo. The mortality rate was twofold greater in the placebo group. Other short-term clinical outcomes and serum levels of malondialdehyde, a marker of oxidative stress, were not significantly different. Importantly, the authors did not report any adverse effects associated with the use of pentoxifylline [18]. The authors concluded that when combined with therapeutic hypothermia, pentoxifylline may be beneficial for treating HIE in near- and full-term neonates.

The results of the ongoing international multicentre trial (PROTECT: ACTRN12616000405415p) of intravenous pentoxifylline in neonates with sepsis or NEC will assist in the design of clinical trials of this drug in neonatal HIE.

Assessing the role of the active metabolites of pentoxifylline is important in studies evaluating its efficacy as a treatment for HIE. The availability of microvolume and dried blood spot analysis via ultrahigh-performance liquid chromatography allows for simultaneous measurement of pentoxifylline and its three metabolites M1 (lisofylline), M4 and M5, making it feasible to include this assessment in such studies. [45]. Evaluating the compatibility of intravenous pentoxifylline with other concurrently administered solutions in preclinical studies is essential from a translational point of view [46].

## Conclusions

Current evidence from a rat model of this condition indicates that pentoxifylline may improve mortality and reduce organ injury following HIE. The evidence in total is adequate to proceed for a robust pilot RCT in human infants with HIE to guide further research in this field. The importance of such studies cannot be overemphasised considering the global burden of HIE and the urgent need for standalone or adjuvant treatments for this condition.

## Data Availability

Data are available upon reasonable request.

## Abbreviations

HIE: Hypoxic ischemic encephalopathy
HICs: High-income countries
LMICs: Lower- and middle-income countries
TH: Therapeutic hypothermia
PRISMA: Preferred reporting items for systematic reviews and meta-analysis
ORs: Odds ratios
RRs: Risk ratios
CI: Confidence interval
ROB: Risk of bias
LOS: Late onset sepsis
NEC: Necrotizing enterocolitis
